# Alpha-1 Antitrypsin—A Target for MicroRNA-Based Therapeutic Development for Cystic Fibrosis

**DOI:** 10.3390/ijms21030836

**Published:** 2020-01-28

**Authors:** Alison M.D. Hunt, Arlene M.A. Glasgow, Hilary Humphreys, Catherine M. Greene

**Affiliations:** Department of Clinical Microbiology, Royal College of Surgeons in Ireland, Dublin 9, Ireland

**Keywords:** cystic fibrosis, neutrophil elastase, antiproteases, alpha-1 antitrypsin, microRNA

## Abstract

Cystic fibrosis (CF) is an autosomal recessive genetic disorder arising from mutations to the cystic fibrosis transmembrane conductance regulator (*CFTR*) gene. Disruption to normal ion homeostasis in the airway results in impaired mucociliary clearance, leaving the lung more vulnerable to recurrent and chronic bacterial infections. The CF lung endures an excess of neutrophilic inflammation, and whilst neutrophil serine proteases are a crucial part of the innate host defence to infection, a surplus of neutrophil elastase (NE) is understood to create a net destructive effect. Alpha-1 antitrypsin (A1AT) is a key antiprotease in the control of NE protease activity but is ineffective in the CF lung due to the huge imbalance of NE levels. Therapeutic strategies to boost levels of protective antiproteases such as A1AT in the lung remain an attractive research strategy to limit the damage from excess protease activity. microRNAs are small non-coding RNA molecules that bind specific cognate sequences to inhibit expression of target mRNAs. The inhibition of miRNAs which target the *SERPINA1* (A1AT-encoding gene) mRNA represents a novel therapeutic approach for CF inflammation. This could involve the delivery of antagomirs that bind and sequester the target miRNA, or target site blockers that bind miRNA recognition elements within the target mRNA to prevent miRNA interaction. Therefore, miRNA targeted therapies offer an alternative strategy to drive endogenous A1AT production and thus supplement the antiprotease shield of the CF lung.

## 1. Introduction

Cystic fibrosis (CF) is a complex inherited disorder that affects numerous organ systems, principally the respiratory and gastro-intestinal tracts [1]. It is inherited in an autosomal recessive manner, where individuals that are heterozygous for a mutant cystic fibrosis transmembrane conductance regulator allele (*CFTR*) are termed carriers of the disease and are largely asymptomatic; whereas those who carry two mutant *CFTR* alleles have the disease. CF is the most common inherited lethal disorder in the Western population, with up to 30,000 individuals in the United States and 27,000 in Europe having the disease [2].

### 1.1. The CFTR Gene and Protein

The *CTFR* gene is located on the long arm of chromosome 7 at position q31.2. It is approximately 250 kb in length and it contains 27 exons [3]. The CFTR protein is a 1480 amino acid, membrane-bound ion channel which is a member of the adenosine triphosphate (ATP)-binding cassette (ABC) superfamily of transporters [4]. These proteins utilise the energy from ATP hydrolysis to drive the transport of various molecules across the cell membrane, e.g., CFTR facilitates the transport of chloride and bicarbonate ions [5]. CFTR works in conjunction with other ion channels including the epithelial sodium channel (ENaC) and the calcium activated chloride channel, anoctamin 1 (ANO1), to regulate fluid movement through the epithelium [6,7]. CFTR is therefore a critical regulator of the volume, pH and mucus viscosity of the airway surface liquid [8]. 

Mutations in the *CFTR* gene lead to a dysfunctional CFTR protein. Currently over 2000 mutations have been described and are divided into seven classes (I to VII) according to their effect on CFTR and therefore the disease severity and presentation [9]. Classes I to VI result in defective protein synthesis, impaired protein trafficking, reduced channel open probability, defective ion channel conductance, decreased membrane expression of CFTR, or decreased CFTR stability, respectively. Class VII is a complete absence of *CFTR* mRNA caused by very large deletions [10]. The most common CFTR mutation is a three base-pair deletion of phenylalanine 508 (F508del) (Class II) and affects 85% of patients [10]. 

### 1.2. CF Disease Presentation

The CFTR protein is expressed in exocrine tissues throughout the body, creating a multisystem presentation of the disease. A defective CFTR protein leads to diminished anion secretions across secretory epithelia, resulting in thickened and viscous mucus in the lung, gastro-intestinal tract and the reproductive system [11]. Although CF is a systemic disease, progressive lung disease remains to be the major contributor of morbidity and mortality to most patients [11].

CF lung disease is attributable to a combination of impaired mucociliary clearance as a consequence of abnormally viscous secretions and by a failure of the innate immune system to clear infections. These factors make the CF airway susceptible to primary and recurrent bacterial infections, blockage, lung inflammation and chronic bacterial infections [11].

Chronic and/or recurrent lung infections leave the lung in a continued pro-inflammatory state, resulting in the development of bronchiectasis. Bronchiectatic airways lose their cartilaginous support, become ‘floppy’ and collapse easily, further impairing mucociliary clearance and predisposing the lung to infection [11]. Over time, chronic mucus plugging and infection damage the airways to such an extent that progressive respiratory failure ensues [12]. 

Numerous advances have been made in the treatment of CF, especially in the clearance of airway infections. However, with the emergence of multi-drug resistant pathogens, fresh challenges now lie ahead in the treatment and management of CF and there is a need for additional therapies for CF lung infection [13].

## 2. Cells of the Innate Immune System

Following pathogen recognition, immune cells are rapidly recruited towards the site of infection in response to the release of pro-inflammatory cytokines and chemokines [14]. Phagocytosis of microbes is highly reliant on neutrophils and macrophages whereas antigen presentation to the adaptive immune system is reliant on macrophages and dendritic cells [14]. Monocyte/macrophage and neutrophil dysfunction are both known to be implicated in CF.

### 2.1. Monocytes and Macrophages in CF 

The notion that CF macrophages are defective has been well established through numerous studies over the past couple of decades. It was found that reduced CFTR expression or CFTR inhibition on macrophages results in hypersecretion of pro-inflammatory cytokines [15]. This may in part be due to the increased expression of pattern recognition receptors (PRRs), such as Toll-like receptor 4 (TLR4) on CF macrophages [16], but it may also be due to the defective autophagy of PRRs serving to further stimulate the pro-inflammatory pathways [17]. Additionally, inflammatory mediators present in the CF lung, including proteases, may chronically activate macrophage PRRs (and also in other cell types), serving to optimise pathogen recognition whilst promoting inflammation [18].

Human and animal studies have also suggested that CF macrophages are intrinsically hyper-inflammatory, with increased production of pro-inflammatory cytokines in response to exposure to various bacteria, pathogen-associated molecular patterns (PAMPs) and inflammatory mediators [17,19]. In addition, studies have shown that negative regulation of pro-inflammatory pathways is impaired in CF macrophages, further establishing a chronically inflamed lung environment [18].

Adding to their hyper-inflammatory qualities, CF macrophages have been shown to have defective bacterial clearance capabilities. Reduced CFTR expression in monocytes impairs β-1 and β-2 integrin-mediated adhesion and chemotaxis, preventing adequate monocyte transmigration to the site of infection [20]. Additionally, diminished CFTR expression on CF macrophages has been shown to hinder their ability to kill phagocytosed bacteria intracellularly. In the CF lung, bacterial clearance capabilities of monocytes or macrophages are further impaired [15]. It was proposed that the absence of CFTR protein is associated with the alkalisation of the phagosome lumen, which impairs *P. aeruginosa* killing [21]. The CF lung has a high protease burden and proteases are known to cleave cell-surface immune receptors [22]. As a result, the ability of a monocyte or macrophage to recognise and clear infections is impaired [18]. Furthermore, cleavage of surface immune receptors has negative consequences for the adaptive immune response to infection [18]. Lastly, it is speculated that CF macrophages may be defective at releasing bactericidal mediators, such as carbon monoxide and antimicrobial enzymes, further impairing bacterial clearance [18].

### 2.2. Neutrophils in CF

Neutrophils are the most abundant immune cell, accounting for approximately 60% of circulating leukocytes [23]. They contain primary (or azurophilic) granules, secondary granules and tertiary granules. Azurophilic granules contain hydrolases, myeloperoxidase (MPO) and neutrophil elastase, which can either target phagocytosed microbes in the phagolysosome or be uncontrollably released into the extracellular space at the site of infection [24]. Secondary granules contain antimicrobial proteins such as lactoferrin and cathelicidin [24]. Tertiary granules contain matrix metalloproteinase-9 (MMP-9), which is involved in the transmigration of neutrophils and in proteolytic degradation of the extracellular matrix [24,25]. Neutrophils release DNA structures that contain neutrophil-derived proteases [26]. These structures, termed neutrophil extracellular traps (NETs), degrade virulence factors and kill bacteria [26]. NETs account for a significant proportion of DNA found in mucus in the CF lung [26]. Furthermore, there is evidence that CF neutrophils may inherently be defective [27]. CF is characterised by recurrent and chronic bacterial infections and the CF lung is dominated by neutrophilic inflammation. Despite their protective role, the chronic presence of neutrophils in the CF lung can lead to irreversible damage to the lung parenchyma through their continued release of proteolytic enzymes [28]. 

## 3. Proteases

There are five classes of proteases present in mammals: serine, cysteine, metallo, aspartic and threonine proteases, with the first four classes being the most prevalent in the human lung [29]. Proteases were originally considered to have solely proteolytic properties. However, a growing body of research has uncovered their involvement in a series of intracellular and extracellular regulatory processes, including tissue remodelling, mucin expression, bacterial killing and neutrophil chemotaxis [29]. With respect to the lung, proteases are secreted from bronchial epithelial cells and are also expressed in monocyte, lymphocyte and granulocyte cell lineages, especially in neutrophils [30]. Proteases cleave extracellular matrix (ECM) proteins, antimicrobial peptides (AMPs) and antiproteases, and they have implications in the immune response to infection. In the healthy lung, proteases are involved in homeostasis and are contributory towards regeneration and repair processes [29]. Elevated protease levels are associated with a large spectrum of pulmonary diseases, including idiopathic pulmonary fibrosis (IPF), emphysema, respiratory tract infections and CF [28]. A number of proteases are expressed in the lung, such as cathepsin G (CatG) and MMPs [28]. However, neutrophil elastase (NE) is a major protease released in the CF lung [31], and its implications in CF will now be discussed further.

### Neutrophil Elastase

NE is a 29 kDa serine protease that is derived from the “elastase, neutrophil expressed” (*ELANE)* gene, found on chromosome 19 at position p13.3 [32]. NE is stored in neutrophil azurophilic granules at a mean concentration of 5.33 mM [33], and is released during neutrophil degranulation, NET formation or during neutrophil necrosis [31]. Despite its name, NE not only degrades elastin, it also degrades most ECM proteins and some plasma proteins, such as collagen (types I-IV), laminin, fibronectin and proteoglycans [34]. NE can also cleave coagulation factors, other proteases and antiproteases, leading to their activation or loss of function [28,35,36].

**Figure 1:**
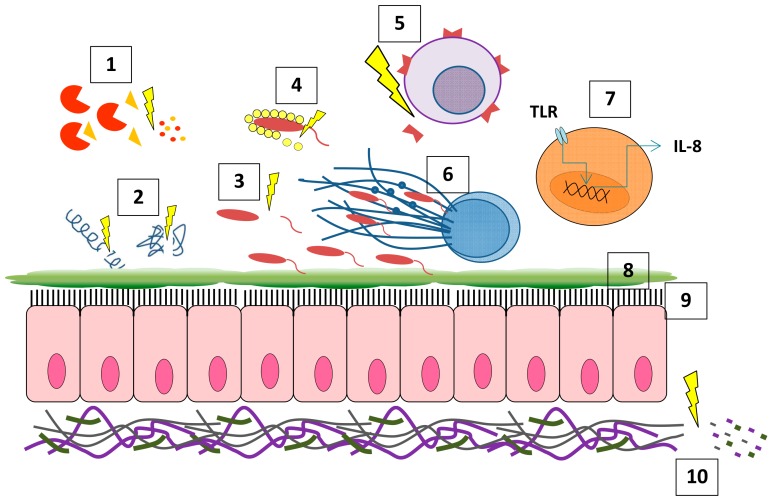
Neutrophil elastase (NE) has a wide range of effects including: (1) cleavage of other proteases/antiproteases; (2) cleavage of antimicrobial peptides (AMPs); (3) cleavage of flagellin; (4) cleavage of opsonizing peptides; (5) cleavage of cell surface receptors; (6) neutrophil extracellular trap (NET) formation; (7) upregulation of pro-inflammatory cytokines; (8) upregulation of mucin genes; (9) impairing ciliary function; (10) degradation of extracellular matrix (ECM) proteins.

Whilst NE’s proteolytic properties contribute to structural destruction of the airways, it is also involved in a number of other processes such as inflammation and the host response to infection (Figure 1). NE upregulates pro-inflammatory cytokine secretion, for example, it upregulates IL-8 secretion through indirect stimulation of TLR4 or TLR2 and their signalling cascades [37,38,39]. This leads to further neutrophil recruitment, generating a continuous and destructive cycle of neutrophilic inflammation and protease release [40]. Interestingly, this effect is reversed when NE activity is inhibited by the serine protease inhibitor, phenylmethylsulfonyl fluoride [40]. Much like other proteases, NE can also impair the innate and adaptive immune system’s ability to clear infections. NE impairs mucociliary clearance by causing mucus hypersecretion via upregulating the expression of mucin genes such as *MUC5AC* and *MUC5B* [41]. It was also shown to upregulate *MUC4* and *MUC1*, however, their roles in the lung are much less understood [42,43]. In addition to enhancing mucus secretion, NE also impairs ciliary function by degrading cilia and reducing their beat frequency [44]. Altogether, the combination of mucus hypersecretion and ciliary dysfunction leads to mucus plugging and an increased predisposition to infection in CF individuals.

NE is necessary for pathogen clearance, however, it can also impair the innate immune system’s ability to resolve infections. It is essential for maximum clearance of intracellular Gram-negative bacteria by neutrophils [45]. Additionally, NE, along with other serine proteases form NETs, which entrap and kill Gram-positive and Gram-negative bacteria [26]. NE was shown to regulate NET formation, with NE knockout mice displaying the inability to form NETs in a *Klebsiella pneumoniae* infection model [46].

In contrast to NE’s infection-resolving abilities, excess NE was shown to cleave and downregulate flagella, an important PAMP found on CF-associated pathogens, such as *P. aeruginosa* [47,48]. Flagella cleavage reduces the innate immune system’s ability to detect these pathogens via the TLR5 signalling pathway [47]. NE can disrupt the complement signalling and clearance pathways by cleaving opsonising peptides, such as C3b and the cell surface receptor C5a [49,50]. It can also cleave macrophage apoptotic cell receptors such as CD36, impairing their ability to clear apoptotic cells [51]. Lastly, NE was shown to cleave AMPs, such as β-defensins and lactoferrin [52,53]. In addition to its effects on the innate immune system, NE can also affect the adaptive immune system. Studies have shown that NE cleaves T-cell receptors, CD2, CD4, CD8 and CD14, impairing monocyte activation, antigen presentation and dendritic cell maturation [54,55]. Despite its original intention for resolving infections, excess NE secretion may in fact be detrimental for pathogen clearance, allowing for increased bacterial survival rates and the presence of recurrent and chronic infections in CF individuals.

## 4. Antiproteases

In the healthy lung, proteases and antiproteases maintain a homeostatic balance, preventing any potential lung damage that may arise from proteases. CF lung disease is dominated by chronic and recurrent infections that are characterised predominantly by neutrophilic inflammation [56]. In response to infection, neutrophils not only phagocytose microbes, they also release an abundance of proteases into the extracellular space, damaging surrounding structures if this process is uncontrolled or excessive [31,56,57]. An imbalance in the protease/antiprotease balance is heavily implicated in CF pathophysiology [28]. Several antiproteases are expressed in the lung [40], and the three major serine antiproteases are elafin, secretory leukocyte protease inhibitor (SLPI) and alpha1-antitrypsin (A1AT).

The CF lung is a protease-rich environment and this protease burden overwhelms the antiprotease capabilities of its principle serine antiprotease, A1AT, leading to a protease-antiprotease imbalance, further damaging the CF lung. Despite the apparent deficit in A1AT function, CF individuals actually secrete increased levels of A1AT and other antiproteases [58]. However, excessive serine protease activity, especially due to NE, leads to complex formation with and enzymatic cleavage of A1AT and other antiproteases such as SLPI and elafin, rendering them inactive [59,60,61].

### 4.1. Elafin

The *elafin* gene codes for a 117 amino acid protein containing a signal peptide, enabling the secretion of a 95 amino acid precursor protein known as pre-elafin or trappin-2 [62]. This precursor is cleaved by the enzyme tryptase to yield mature elafin—a small 6 kDa, 57 amino acid peptide [62,63]. Elafin is found constitutively expressed at many epithelial surfaces including the bronchial and alveolar cells of the lung, and in immune cells such as neutrophils and macrophages [64,65,66,67,68]. Elafin is a potent inhibitor of NE and proteinase-3 (PR3) [69,70]. Elafin and trappin-2 exert antibacterial and antifungal effects independent of antiprotease activity [71,72]. Antiviral activity has also been reported [73]. Additionally, elafin inhibits LPS-induced activation of the pro-inflammatory nuclear factor kappa-light-chain-enhancer of activated B cells (NF-κB) and activator protein 1 (AP-1) pathways [74].

### 4.2. SLPI

SLPI is an 11.7 kDa serine protease inhibitor that is comprised of 107 amino acid residues [75,76]. It inhibits a number of proteases, including NE, CatG, trypsin, chymotrypsin, chymase and tryptase [77]. SLPI demonstrates a wide range of expression. It is expressed by a number of mucosal surfaces and is secreted by various cell types including neutrophils, macrophages and epithelial cells of the respiratory and gastro-intestinal tracts [29]. Specifically in the lung, SLPI is secreted by the club cells and goblet cells of the respiratory epithelium; by the serous cells in submucosal glands; and by alveolar macrophages [78]. SLPI’s antiprotease functions are particularly important in the upper airways [60]. SLPI also has antimicrobial, anti-inflammatory and immunomodulatory activity [79,80,81].

### 4.3. A1AT

A1AT is an acute-phase 52 kDa glycoprotein that is primarily synthesised and secreted by liver hepatocytes and circulates systemically. Additionally, it is secreted in smaller quantities by neutrophils, monocytes and enterocytes, and by alveolar macrophages and bronchial epithelial cells in the lung [82]. A1AT is present in all tissues around the body and it most notably inhibits NE, however it also inhibits a wide range of other proteases, including CatG, PR3, trypsin, chymotrypsin, plasmin, thrombin and factor Xa, amongst others [29]. A1AT was also shown to improve bacterial clearance by preventing the cleavage of neutrophil surface complement receptors (e.g., CD35) and C-X-C Motif Chemokine Receptor 1 (CXCR1) receptors [83]. The serum concentration of A1AT in a healthy individual is 85–250 mg/dL, showing a three- to five-fold increase during inflammation and injury [84].

## 5. Current Treatment of CF Lung Disease

The treatment of CF lung manifestations can be divided into various categories: (1) airway clearance therapies; (2) bronchodilators; (3) anti-inflammatory therapies; (4) infection prophylaxis and treatment; and (5) CFTR modulators; these have been extensively reviewed elsewhere [85]. A number of experimental and established antiprotease therapies are also possible. CF patients with advanced pulmonary disease may also receive lung transplants and/or respiratory support [86].

## 6. Novel Therapies for CF Lung Disease

### 6.1. Antiprotease Therapies

The CF lung is a protease-rich environment, and this protease burden overwhelms the protective abilities of endogenous antiproteases, placing the CF lung at greater risk for irreversible airway destruction. This protease/antiprotease imbalance largely contributes towards CF lung pathology [87], prompting various research groups to investigate the therapeutic potential of antiproteases. Antiprotease therapies for CF can be divided into two distinct categories: antiprotease augmentation therapies and pharmacological protease inhibition [87].

In the early 1990s, McElvaney et al. investigated the effects of A1AT augmentation therapy in CF patients through two different routes of administration: intravenously and aerosolized [58]. Their initial study investigated the effects of intravenous administration of A1AT at doses ranging from 60 to 120 mg/kg in CF patients. They noticed that only the 120 mg/kg dose was sufficient to suppress the effects of NE. However, the effects were transient, with the re-emergence of active NE in bronchoalveolar lavage fluid (BALF) within one week of A1AT administration [58]. The same group subsequently investigated the effects of administering higher doses of aerosolised A1AT to CF patients, ranging from 1.5 to 3 mg/kg A1AT twice daily for one week. Aerosolised administration of A1AT resulted in inhibition of NE and re-established anti-NE activity on the respiratory epithelium, but only if the A1AT level was above a certain threshold of 8 µM in BALF [58]. The authors concluded that despite the therapeutic potential A1AT augmentation therapy offers, the marked variability of NE levels in the CF lung makes it difficult to determine an optimal dosage for A1AT administration [58]. In contrast to the anti-NE effects observed in the previous studies, another study found that inhaled A1AT augmentation therapy did not substantially decrease anti-NE activity in CF induced sputum samples [58]. However, these discrepancies may have possibly been due to the use of different aerosol devices across the two studies and also to the biological samples used to examine the antiprotease effects of inhaled A1AT therapy (BALF vs. induced sputum) [58,88].

Lately, much work has gone into developing novel protease inhibitors with the aim of improving their resistance against protease-mediated inactivation, in the hope of enhancing their antiprotease capabilities. This is achieved either through modifying pre-existing drugs/proteins, or by generating synthetic protease inhibitors. Initial studies investigated the use of recombinant forms of endogenous antiproteases, such as A1AT, SLPI and human monocyte/neutrophil elastase inhibitor (hMNEI) [89,90,91,92]. It was found that aerosolised administration of these recombinant antiproteases reduced the release of a variety of proteases, pro-inflammatory mediators, and in the case of recombinant hMNEI, enhanced bacterial clearance in murine models infected with *P. aeruginosa* [89,90,91,92]. Subsequent studies have also investigated the therapeutic potential of functional variants of the endogenous antiproteases, elafin and SLPI, and found that these variants retained their antiprotease properties, blunted the pro-inflammatory response to infection in the lung, and showcased an increased resistance to protease-mediated degradation [93,94].

A number of novel synthetic protease inhibitors have been identified for potential use in CF, with POL6014, DX-890, KRP-109, and AZD9668 being examples. POL6014 significantly reduces NE activity without impairing neutrophil function [95] while DX-890 decreases pro-inflammatory cytokine secretion and neutrophil transmigration [96]. KRP-109 may possibly reverse mucus plugging in the CF airways [97]. However, a study by Yamada et al. investigating the effects of KRP-109 administration in murine pneumococcal models found that there was no difference in BALF NE activity between KRP-109-treated mice and controls [98]. Nonetheless, they noticed that KRP-109 mice had higher survival rates, and histologic examination revealed reductions in alveolar inflammation [98]. AZD9668 underwent phase 2a randomised-controlled, parallel-group clinical trials where patients either received oral AZD9668 or placebo for 4 weeks [99]. AZD9688 was well-tolerated and led to reductions in free and total urinary desmosine—biomarkers of elastin degradation [99]. However, there were no observable differences in sputum NE activity, lung function, respiratory symptoms, quality of life, or use of reliever medication between both treatment arms [99]. Lastly, 2-O, 3-O-desulfated heparin (ODSH), doxycycline and N-Arylacyl O-sulfonated aminoglycosides were shown to exhibit antiprotease properties [100,101,102,103]. A summary of the findings from these various therapeutic antiprotease studies is outlined in Table 1.

### 6.2. A1AT Gene Therapy

An emerging area of research is A1AT gene therapy. Much of the published literature is directed towards A1AT deficiency (A1ATD); however, its findings may be applicable to CF given the similarities between the two diseases. Various studies investigated the effects of wild-type human *A1AT* gene (M-A1AT) transfer using retroviral and adenoviral vectors, and they showcased successful M-A1AT transfer, M-A1AT synthesis and secretion, although the effects were short-lived [105]. It was also noted that both these vectors had associated side effects. Retroviral vectors were associated with hypotension, lethargy, hematemesis and death, which was observed in experiments using dog models [106]. It is important to note that retroviral gene transfer was shown to be associated with an increased risk of mutagenesis, which causes neoplastic transformation [107]. Adenoviral vectors, on the other hand, were shown to be highly immunogenic [108]. As a way to combat the negative side effects of retroviral and adenoviral therapies, researchers investigated the use of recombinant adeno-associated viral vectors (rAAV) in M-A1AT therapy. rAAVs have become the gold-standard in gene therapy because of their low pathogenicity and immunogenicity [105]. Unlike retroviruses, they do not integrate in their host cell’s genome, which removes the risk of insertional oncogenesis [105]. rAAVs do exhibit immunogenic properties, such as the formation of neutralising antibodies, but they do not activate cytotoxic T-cells [105]. Furthermore, rAAVs can also infect and persist in both dividing and non-dividing cells for long periods of time, allowing for prolonged gene expression [105]. Since rAAVs have low pathogenicity and offer prolonged, gene expression, it has long been the focus of M-A1AT augmentation therapy research.

RNA interference (RNAi) is another potential approach to A1AT therapy. In contrast to A1AT augmentation therapy, RNAi is involved in the post-transcriptional silencing of proteins [109]. One study investigated the effects of post-transcriptional silencing of mutated A1AT (Z-A1AT) by incorporating microRNAs into rAAVs [110]. The effects on microRNA-mediated silencing of Z-A1AT came into question following the realisation that M-A1AT augmentation therapy failed to reduce Z-A1AT expression in tissues [110]. Increased Z-A1AT expression results in the accumulation of mutant protein in hepatocytes, predisposing them to damage [110]. It was found that dual microRNA and rAAV-based therapy resulted in long-term knockdown of circulating Z-A1AT [110]. In addition, in vivo experiments showcased a significant and widespread decrease in Z-A1AT accumulation within hepatocytes [110]. These findings suggest that dual microRNA and rAVV therapy has the potential to halt the progression of A1ATD-mediated liver disease. Although this study does not investigate the effects of microRNA-based gene therapies in the lung, it does serve to highlight its potential for the treatment of CF lung disease.

## 7. microRNAs

microRNAs (miRNAs or miRs) are short non-coding RNAs that are around 20–25 nucleotides long [111]. They are generated in a step-wise process and they negatively control gene expression through translational repression and/or degradation of its target messenger RNA (mRNA) [112,113]. This is accomplished through their sequence-specific hybridisation with miRNA responsive elements (MREs) in the 3’ untranslated region (3’UTR) of target mRNAs. miRNAs were first described in the early 1990s by Lee et al. in the nematode, *Caenorhabditis elegans* [114], however the term “miRNA” was not coined until the early 2000s. Ever since their discovery, much research has gone into miRNAs and their roles in developmental biology and other biological processes, including proliferation and apoptosis [115]. Aberrant miRNA expression was shown to be implicated in a number of physiological and pathological processes such as in ageing, cancer and autoimmune disease [116,117,118]. In vitro modulation of miRNAs can be achieved using inhibition or augmentation strategies. Artificial overexpression of miRNAs can be achieved by transfection of miRNA mimics or genes expressing miRNAs into target cells [119]. One commonly used method to inhibit miRNA experimentally is the use of locked nucleic acid cholesterol-tagged antisense oligonucleotides termed antagomirs [120]. Recently, several research groups have explored the role of miRNAs in CF, and their findings will be outlined below.

### 7.1. miRNAs in CF

There are several miRNAs known to regulate CFTR expression, e.g., miR-145, miR-494, miR-223 and miR-509-3p [121,122,123,124]. Each of these has been found upregulated in primary bronchial brushings and/or airway epithelial cells from the CF lung [122,123]. miR-138 can also modulate CFTR expression through the influence of its mRNA target, the transcriptional regulator SIN3A [125]. The calcium-activated chloride ion channel, ANO1, has also been shown to contribute to CF pathology and is downregulated in CF epithelium [126]. ANO1 is targeted by miR-9, which is increased in fully differentiated bronchial epithelial cells from CF patients [127].

Various studies have shown that miRNA dysregulation has implications in the innate immune response of CF individuals. For example, miR-126 is downregulated in CF bronchial brushings, and its target mRNA, target of Myb1 (TOM1; a negative regulator of the TLR2/4 pathway), is reciprocally upregulated [128]. In contrast, another negative regulator of the TLR4 pro-inflammatory cascade, caveolin 1 (CAV1), is reduced in CF macrophages due to increased levels of miR-199a-5p [129].

IL-8 is a major neutrophil chemokine that is present at elevated levels in the CF lung. miR-17 and miR-93 directly target IL-8 expression, and both miRs are decreased during *P. aeruginosa*-induced inflammation in CF bronchial epithelial cells [130,131]. Increased levels of miR-155 in CF further contribute to increased IL-8 by decreasing the target mRNA, Src homology-2 domain-containing inositol 5-phosphatase 1 (SHIP1), as this enables stabilisation of the IL-8 mRNA transcript [132]. In contrast, miR-146a is over-expressed in CF macrophages and is suggested to limit the inflammatory response through its targeting of IL-6 [133].

In contrast to reduced miR-17 levels in CF bronchial brushings, miR-17 is upregulated in CF macrophages, where it targets key autophagy pathway molecules ATG7 and ATG16L1 [134]. As well as dysfunctional autophagy, CF macrophages also display impaired phagocytosis. CF macrophages have increased miR-181b and thus lower levels of its target, a cell surface receptor recognised by lipoxin A4 [135]. Inhibiting miR-181b restores lipoxin A4-induced phagocytic ability [135].

As well as targeting SHIP1, miR-155 also controls the expression of regulatory-associated protein of mTOR, complex 1 (RPTOR) [136]. This links the increased miR-155 levels in CF epithelial cells to the progressive fibrosis of the CF lung, as RPTOR inhibition activates the transforming growth factor beta (TGFβ) pathway to induce fibrosis [136]. Ongoing destruction of healthy lung tissue in CF is also partly mediated by proteases. The elastolytic protease, cathepsin S, is increased in CF BALF and correlates with poorer lung function [137]. miR-31, decreased in CF bronchial brushings, targets the transcription factor interferon regulatory factor 1 (IRF-1), thereby regulating cathepsin S expression [137].

Finally, miRNAs also contribute to CF pathology via an effect on the unfolded protein response (UPR), a network of signalling pathways activated due to the endoplasmic reticulum (ER) stress arising from misfolded CFTR. miR-221 is increased in CF bronchial brushings, whilst its target, activating transcription factor 6 (ATF6; a transcription factor involved in the UPR), is decreased [138]. The role of dysregulated miRNAs in CF and other lung diseases has been more extensively reviewed elsewhere, for example Glasgow et al. [139] and Dutta et al. [140].

### 7.2. miRNA Therapeutics

Abnormally expressed mRNAs or proteins associated with disease represent therapeutic targets for miRNA-based drug development. Likewise, the same strategies can be employed if disease-associated miRNAs are to be targeted (Table 2). 

For example, in order to suppress the expression of an overly abundant disease-associated miRNA an inhibition approach may be adopted; conversely, to restore expression of a down-regulated miRNA, a miRNA mimic delivery strategy can be employed [141]. In CF, where A1AT levels and activity are impaired for a variety of reasons, as discussed, an inhibition approach focusing on miRNAs that regulate the 3’UTR of the *SERPINA1* mRNA would appear to be worth considering. In theory, this should inhibit the target miRNA(s) and in turn increase expression of *SERPINA1* mRNA and protein. Two major approaches are possible—antagomirs or target site blockers (TSBs, also known as ‘masks’). Antagomirs are chemically modified synthetic antisense oligonucleotides which are complementary to a target miRNA. Once delivered inside a cell, an antagomir can bind to and sequester its target miRNA, thereby preventing it from negatively regulating all of its target mRNAs. As such, the effects of antagomirs can have substantial impact on pathways wherein the target miRNA affects different parts of the same biological process. However, this overall approach could have undesired off-target effects on other genes that are not part of the targeted pathway. A more precise method that may be required in other specific instances is the use of a TSB. In this approach, a locked-nucleic acid antisense oligonucleotide is custom-designed to compete specifically with a miRNA for binding to an individual miRNA recognition element within the 3’UTR of a target mRNA, hence preventing it from gaining access to those sites. 

By their nature, nucleic acid-based therapies are prone to degradation by nucleases but also may activate the innate immune system. Therefore, delivery of miRNA-targeted therapeutics is a challenge. Many physiological barriers need to be overcome in order for the therapy to reach its site of action within a specific cell and to ensure it retains its activity whilst remaining invisible to the immune system. Optimising these processes is the focus of multiple cell biology, pharmaceutics and drug development projects. TargomiR is an intravenous miRNA mimic therapy for solid tumours in malignant pleural mesothelioma. The therapy is based on miR-16-loaded minicells and is a miRNA delivery therapy designed to restore the loss of miR-16 evident in malignant pleural mesothelioma patients. A Phase 1 open label dose-escalation study of TargomiR demonstrated tolerability and early signs of antitumour activity [142]. miRNA inhibition therapies for lung disease have yet to progress to first-in-human studies. Recent and ongoing work in this group is focused on the identification of novel miRNAs that specifically regulate A1AT expression in monocytes or hepatocytes that could form the basis of miR-medicines targeting the protease/antiprotease imbalance in the CF lung, either locally or systemically [143].

## 8. Conclusions

As CF lung disease is so dominantly characterised by neutrophilic inflammation, the neutrophil serine protease NE is present at vastly excessive levels. In addition to causing damage by degrading its target extracellular matrix substrates, NE can aggravate lung inflammation. A1AT is the most abundant inhibitor of NE in the airways. However, the increased number of neutrophils in CF leads to a gross imbalance between NE activity and A1AT defences. Therapeutic enhancement of pulmonary A1AT could be a potential intervention to aid the control of NE-mediated destruction during CF exacerbations. As an alternative to the delivery of recombinant A1AT protein to the lung and its inherent caveats, a novel approach would be the delivery of molecules that drive A1AT expression in the patient’s own cells e.g., microRNA-targeted therapies.

## Figures and Tables

**Table 1:** Antiprotease therapies in cystic fibrosis (CF).

Therapy	Main Findings	Study(s)
**A1AT Augmentation Therapies**		
Intravenous A1AT	Transiently suppressed the effects of NE in the lungs of CF patients	McElvaney, et al. [58]
Inhaled, aerosolised A1AT	Inhibited NE in the respiratory epithelium of CF patients (if A1AT BALF levels reached >8 µM)	McElvaney et al. [58]
	Administration to CF patients did not substantially decrease anti-NE activity in induced sputum samples	Cantin et al. [104]
**Recombinant Antiproteases**		
Recombinant A1AT (rA1AT)	Administration to CF patients reduced NE/A1AT complex and sputum MPO levels; downward trend of NE activity in sputum did not reach statistical significance	Martin et al. [89]
Recombinant SLPI (rSLPI)	Increased anti-NE activity in the lungs of CF patients; Enhanced antioxidant protection in sheep lung models by raising glutathione levels	Vogelmeier et al. [91]
	Significantly reduced active NE, IL-8 and neutrophil number in epithelial lining fluid (ELF)	McElvaney et al. [92]
Recombinant hMNEI	Significantly reduced inflammatory injury in murine models of *P. aeruginosa* infection; significantly enhanced bacterial clearance in *P. aeruginosa* infected rat lungs	Woods et al. [90]
**Antiprotease Functional Variants**		
Elafin functional variants (“GG”- and “QQ”-elafin)	Both variants showed increased resistance to degradation when incubated with BALF from CF patients. GG-elafin showed enhanced LPS neutralisation in vitro, and decreased inflammatory cell infiltration in a murine model of acute lung injury. The latter was associated with a reduction in monocyte chemoattractant protein-1 (MCP-1).	Small et al. [93]
SLPI functional variants (“SLPI-A16G” and “SLPI-S15G-A16G”)	Both variants showed enhanced resistance to degradation when incubated with sputum from CF patients. SLPI-A16G demonstrated increased anti-inflammatory activity in a murine model of *P. aeruginosa* infection.	Camper et al. [94]
**Synthetic Protease Inhibitors**		
POL6014	Significantly reduces NE activity without impairing neutrophil function	Polverino et al. [95]
DX-890	Decreases pro-inflammatory cytokine secretion and neutrophil transmigration	Dunlevy et al. [96]
KRP-109	May reverse mucus plugging in CF airways by decreasing NE-prompted mucin degradation	Chillappagari et al. [97]
	No difference in BALF NE activity between KRP-109-treated murine pneumococcal models and controls, however KRP-109-treated mice had higher survival rates and reduced alveolar inflammation	Yamada et al. [98]
AZD9688	Reduced free and total urinary desmosine (biomarkers of elastin degradation), but no observable differences in sputum, NE activity, lung function, respiratory symptoms, or use of reliever medication	Elborn et al. [99]
**Modified Drugs and Pre-Existing Drugs**		
2-*O*, 3-*O*-desulfated heparin (ODSH)	ODSH are ineffective in CF sputum in the absence of dornase α (recombinant DNase), as ODSH and DNA compete for NE binding sites. ODSH have a higher potency of NE inhibition than DNA.	Kummarapurugu et al. [100]
	The 2-O and 3-O sulfate groups on heparin can be removed to reduce its anticoagulant activity without impairing its anti-inflammatory activity. ODSH inhibits, NE, CatG, complement activation and binding to P-selectin	Rao et al. [101]
*N*-Arylacyl *O*-sulfonated aminoglycosides (KanCbz and NeoCbz)	KanCbz inhibits NE, PR3 and CatG. NeoCbz inhibits NE and CatG Both KanCbz and NeoCbz protected respiratory epithelial cells from protease-mediated destruction	Craciun et al. [102]
Doxycycline	Doxycycline significantly reduced sputum MMP-9 levels and was associated with a 1.6-fold increase in tissue inhibitor of metalloproteinase-1 (TIMP1) levels	Xu et al. [103]

**Table 2:** The basis of miRNA-targeting therapeutic strategies.

Dysfunction	Overabundant mRNA/Protein	Lack of a mRNA/Protein	Overexpressed MicroRNA	Underexpressed MicroRNA
Strategy	↑ microRNA(s)	↓ microRNA(s)	↓ microRNA	↑ microRNA
Method	miRNA mimic(s)	Antagomir(s) or TSB(s)	Antagomir or TSB(s)	miRNA mimic

↑ = increase; ↓ = decrease.

## Abbreviations

3’UTR	3’ Untranslated region
A1AT	Alpha-1 antitrypsin
A1ATD	Alpha-1 antitrypsin deficiency
ABC	Adenosine triphosphate binding cassette
AMP	Antimicrobial peptide
ANO1	Anoctamin 1
AP-1	Activator protein 1
ATF6	Activating transcription factor 6
ATP	Adenosine triphosphate
BALF	Bronchoalveolar lavage fluid
CatG	Cathepsin G
CAV1	Caveolin 1
CF	Cystic fibrosis
CFTR	Cystic fibrosis transmembrane conductance regulator
CXCR1	C-X-C Motif Chemokine Receptor 1
ECM	Extracellular matrix
ELANE	Elastase, neutrophil expressed
ELF	Epithelial lining fluid
ER	Endoplasmic reticulum
hMNEI	Human monocyte/neutrophil elastase inhibitor
IPF	Idiopathic pulmonary fibrosis
IRF-1	Interferon regulatory factor 1
MCP-1	Monocyte chemoattractant protein 1
miRNA	Micro RNA
MMP-9	Matrix metalloproteinase 9
MPO	Myeloperoxidase
MRE	Micro RNA responsive element
mRNA	Messenger RNA
NE	Neutrophil elastase
NET	Neutrophil extracellular trap
NF-κB	Nuclear factor kappa-light-chain-enhancer of activated B cells;
ODSH	2-*O*, 3-*O*-desulfated heparin
PAMP	Pathogen associated molecular pattern
PR3	Proteinase 3
PRR	Pattern recognition receptor
rAAV	Recombinant adeno-associated viral vector
RNAi	RNA interference
RPTOR	Regulatory-associated protein of mTOR, complex 1
SHIP	Src homology-2 domain-containing inositol 5-phosphatase 1
SLPI	Secretory leukocyte protease inhibitor
TSB	Target site blocker
TGFβ	Transforming growth factor beta
TIMP-1	Tissue inhibitor of metalloproteinase 1
TLR	Toll-like receptor
TOM1	Target of Myb1
TSB	Target site blockers
UPR	Unfolded protein response
